# The Therapeutic Effects of Lycopene Against Experimental Giardiasis in Mice

**DOI:** 10.1002/fsn3.4515

**Published:** 2024-10-23

**Authors:** Jahan Parvaneh, Fatemeh Namazi, Seyed Mostafa Razavi, Saeed Nazifi, Hassan Nayebzadeh

**Affiliations:** ^1^ Department of Pathobiology, School of Veterinary Medicine Shiraz University Shiraz Iran; ^2^ Department of Clinical Studies, School of Veterinary Medicine Shiraz University Shiraz Iran; ^3^ Department of Pathobiology, Faculty of Veterinary Medicine Lorestan University Khorramabad Iran

**Keywords:** antioxidant, *giardia*, lycopene, mice

## Abstract

*Giardia duodenalis* is a protozoan parasite that infects approximately one billion people worldwide. In this study, the effects of lycopene on experimental giardiasis in mice were investigated by evaluating cyst shedding rate, weight changes, duodenal antioxidant status, and histopathological alteration. Ninety‐five male mice aged four to six weeks were divided into six groups of 15 and one group of 5 as the negative control. All mice were infected with 2 × 10^5^ *Giardia duodenalis* (B genotype) cysts except for the negative control. The treatment groups were treated with metronidazole, mixed lycopene‐metronidazole, and 5, 10, and 20 mg/kg lycopene for 7 days. The mice treated with lycopene 20 and mixed lycopene‐metronidazole had no cyst shedding from the 7^th^ day of treatment. The mildest lesions were observed in the mixed lycopene‐metronidazole group. Results showed that treatment with mixed lycopene‐metronidazole significantly increased TAC and decreased MDA levels as compared with the positive control. It seems that the antigiardial effect of lycopene is related to its antioxidant properties. Nevertheless, direct effect of lycopene on *Giardia* and its probable mechanism remain to be investigated.

## Graphical abstract

The therapeutic effects of lycopene against experimental giardiasis were investigated. Lycopene and mixed lycopene‐metronidazole can increase antioxidant capacity against Giardia duodenalis and stop cyst shedding. It seems that the antigiardial effect of lycopene is related to its antioxidant properties.

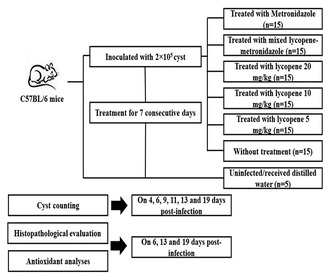

## 1 Introduction

*Giardia duodenalis* is a protozoon, which infects more than 200 million cases in the world annually (Ansell et al. 2015), and has a significant impact on children in developing countries (Ma'ayeh, Knörr, and Svärd 2015). The parasite is transmitted by fecal‐oral method (Goyal, Rishi, and Shukla 2013), and the life cycle consists of two stages: trophozoite and cyst. After swallowing, the cysts turn into trophozoites and they attach to intestinal cells using their adhesive disc and eventually become cysts, excreted in the feces, and this cycle repeats (Einarsson, Ma'ayeh, and Svärd 2016). After a latent period of about one to two weeks, clinical manifestations including watery diarrhea, abdominal pain, nausea, and vomiting occur (Certad et al. 2017). Various mechanisms including damage to epithelial cells, cessation of cell proliferation, T lymphocyte‐induced cell damage, and inactivation of proper disaccharidase enzymes have been suggested for parasite pathogenicity (Bartelt and Sartor 2015).

There are many compounds recommended for treating giardiasis. Despite the beneficial effects of metronidazole as a choice drug, there are increasing reports of side effects such as nausea and a metallic taste (Hill et al. 2005). Furthermore, some treatment failure due to drug resistance has been reported (Amer, Mossallam, and Mahrous 2014). Therefore, it seems that finding new anti‐Giardia drugs is an interesting research topic.

Lycopene, a member of carotenoid family, is a fat‐soluble antioxidant produced by many plants and microorganisms, but animals and humans are not able to produce it (Paiva and Russell 1999). Lycopene stimulates endogenous antioxidative enzymes (Subhash, Bose, and Agrawal 2007). A diet rich in tomato and its products containing lycopene has protective effects against various chronic diseases by reducing oxidative damage (Agarwal and Rao 2000). Lycopene could inhibit inflammatory process by a significant reduction in myeloperoxidase activity, TNF‐α and NO level, and increasing glutathione in rats (El‐Ashmawy et al. 2018). Also, it increased total antioxidant, HDL‐associated paraoxonase 1 function and decreased expression of inflammatory mediators, which led to improve LPS‐induced oxidative stress (Alvi et al. 2017). It has revealed that lycopene could ameliorate hematological and antioxidant parameters in fish (Yonar et al. 2020). A previous study showed the inhibitory effects of tomato peel powder on the growth of three trichomonad strains (Friedman et al. 2021). The main goal of the present study was to evaluate the effects of lycopene in experimental giardiasis.

## 2 Materials and Methods

### 2.1 Preparation of Cyst

Fecal sample from a patient with giardiasis was selected as the source of infection. The cysts were concentrated and purified by flotation method (Buraud et al. 1991). The collected cysts were washed twice in distilled water and centrifuged at 5000 rpm for 5 min. The precipitate was placed in a sterile tube containing 1% sodium hypochlorite for 10 min on an ice bath and then washed three times in phosphate‐buffered saline.

### 2.2 PCR and Sequencing

DNA extraction was performed using the modified cetyltrimethylammonium bromide (CTAB) extraction method for stool samples (Allen et al. 2006).

Polymerase chain reaction (PCR) was done on small ribosomal subunit (18S rRNA) using the primers designed by us, including FORGIAR: 3ˊ‐GAGCAG ATC CTGAAGAACTCCCT‐5ˊ, REVGIAR: 3ˊ‐TCCACTGGAGCCTCACGGA‐5ˊ. PCR for each 25 μL final volume reaction was performed using 12.5 μL RedMaster PCR 2X (Sinaclon, Iran), 1 μL of each primer (10 pM), 4 μL gDNA template, and 6.5 μL ddH2O. PCR reactions were carried out in a thermocycler (BioRad, USA) based on a touchdown temperature profile: 3 min at 94°C, 11× (30 s at 94°C, 30 s at 60°C–50°C, 45 s at 72°C), followed by 24× (30 s at 94°C, 30 s at 50°C, 45 s at 72°C), 10 min at 72°C. The PCR products were visualized with %1 agarose gel electrophoresis, and the desired bands were submitted to a third‐party service provider (Codon Genetic Group, Iran) for sequencing using Applied Biosystems‐ABI, 3130XL.

### 2.3 Phylogenetic Analysis

DNA sequences were manually checked using FinchTV software (www.geospiza.com) to correct any sources of error or ambiguities if present. Homologies with the available sequences data in GenBank were checked using the BLASTn option. Finally, the sequence was submitted to GenBank under accession number OP056331. Then, it was aligned using SeaView4 software (Gouy, Guindon, and Gascuel 2010). We calculated the genetic distances among and between sequences using maximum composite likelihood (MCL) that was modeled in MEGA7 (Kumar, Stecher, and Tamura 2016). To construct the phylogenetic tree, a glutamate dehydrogenase (GDH) alignment sheet was analyzed using BEAST (Ver. 2.6.0) (Bouckaert et al. 2014) based on the Bayesian Inference (BI) method. BI employs Markov Chain Monte Carlo (MCMC) algorithms and infers a most credible tree given the posterior probabilities of alternative tree topologies. GDH sequences including a single sequence of the present study, as well as the comparable Genbank data sequences of the *Giardia* genus (*G. lamblia* and *G. intestinalis*), were used. Sequences were selected according to the similarity revealed by the BLAST algorithm. The phylogenetic trees were summarized and visualized using TreeAnnotator and FigTree (Ver. 1.4.4.), respectively.

### 2.4 Experimental Design

Ninety‐five male C57BL/6 mice aged 4–6 weeks were distributed into six groups of 15 animals and a negative control group of 5. All mice in groups 1–6 were infected with 2 × 10^5^ *Giardia* cysts by stomach tube. On the sixth‐day post‐infection and after confirming the parasite shedding in the fecal sample, the treatment was performed orally by stomach tube for 7 consecutive days. In group 2, each animal received 50 mg/kg metronidazole (Ani Darman Co, Iran). In groups 3–5, each mouse received 5, 10, and 20 mg/kg of lycopene, respectively (Vitabiotics Co, Iran). In group 6, each animal received mixed lycopene‐metronidazole. On 6 (before starting treatments), 13 and 19 days post‐infection, five mice from each group 1–6 were killed humanely, and the duodenal samples, as the predilection site of *Giardia*, were taken for histopathological and biochemical analyses.

### 2.5 Cyst Counting

Cyst counting was done on 4, 6, 9, 11, 13, and 19 days post‐infection and examined by the direct method (Oguoma and Ekwunife 2007). The number of *Giardia* cysts per gram of feces was calculated after the formol–ether concentration technique using the formula *N* = *S*/(*V* × *W*); *N*: number of cysts/g of feces, S: number of counted cysts, *V*: volume of examined sample, *W*: stool weight (g) (El‐Nahas et al. 2013).

### 2.6 Animals’ Weight

Weighting was performed simultaneously with fecal sampling by a digital laboratory scale with 0.01 accuracy.

### 2.7 Histopathological Evaluation

A piece of duodenal samples was collected, fixed in neutral buffered formalin (10%), embedded in paraffin, cut at 5 μm, and stained with hematoxylin and eosin (H&E). The histopathological evaluations were performed by a pathologist blinded to treatment allocation.

### 2.8 Antioxidant Analyses

Sampling and measuring the antioxidant capacity of duodenal tissue were performed simultaneously with histopathological sampling.

TAC concentration was assessed using a commercial kit (ZellBio GmbH, Ulm, Germany) with 0.1 mM sensitivity based on colorimetric changes in the chromogenic substrate (tetramethyl benzidine) at 490 nm (Trachootham et al. 2008).

The commercial kits (Zell Bio GmbH, Germany) were used to measure MDA, and the color complex was calorimetrically measured at 535 nm. The values were expressed as mmol/mg tissue protein.

### 2.9 Statistic Analysis

The statistical analysis of the obtained data was performed using SPSS 22 software, one‐way analysis of variance and Tuckey's test. *p* < 0.05 was considered statistically significant.

## 3 Results

### 3.1 PCR and Sequencing

**FIGURE 1:**
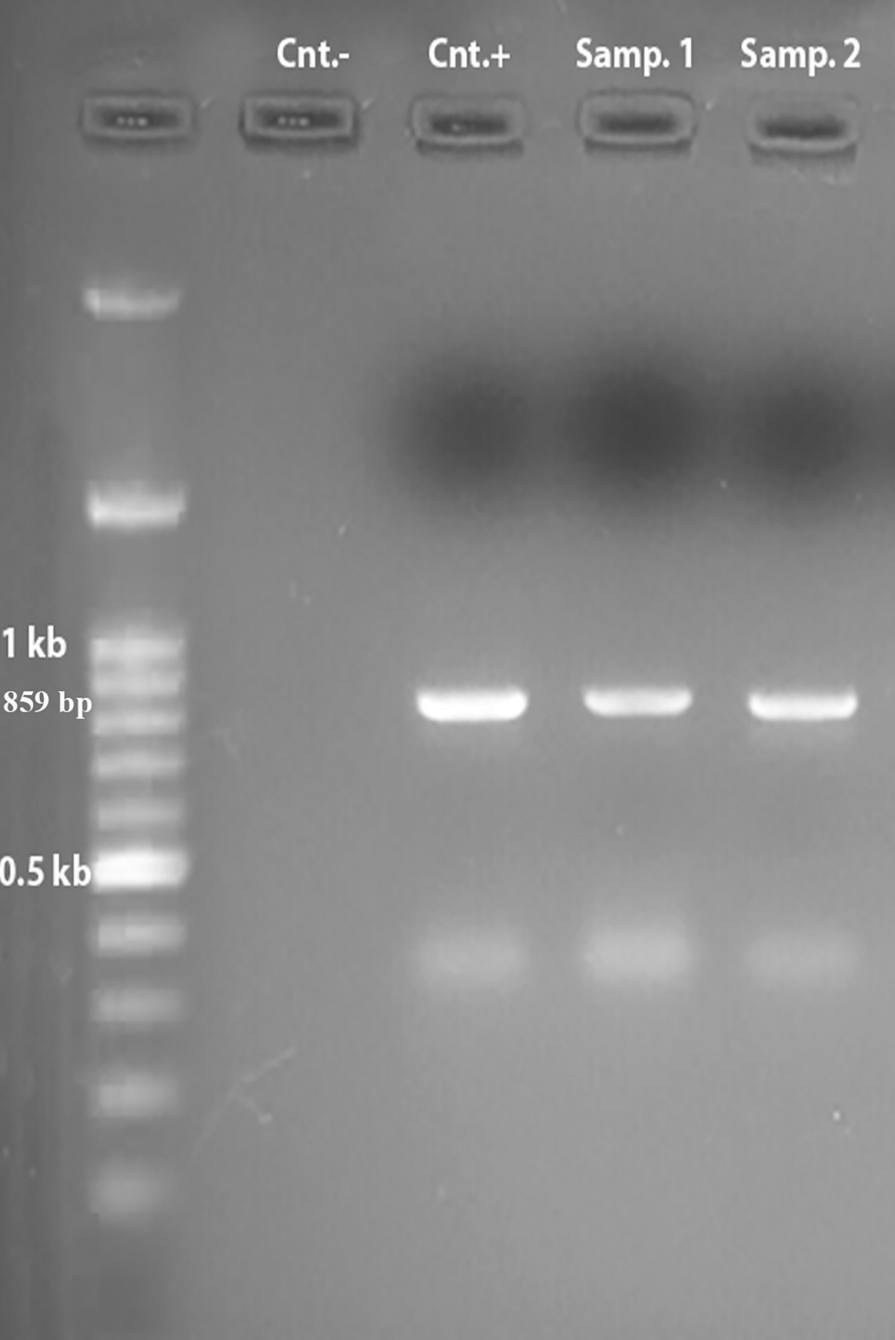
Agarose gel electrophoresis of GDH gene from analyzed *Giardia* isolates. Marker: 100 bp; Cnt.−: Negative control; Cnt.+: Positive control; Samp. 1: Sample 1; Samp. 2: Sample 2 (The size of bands = 859 bp).

An 859 bp fragment of the GDH gene was amplified by PCR (Figure 1).

### 3.2 Phylogenetic Tree

**FIGURE 2:**
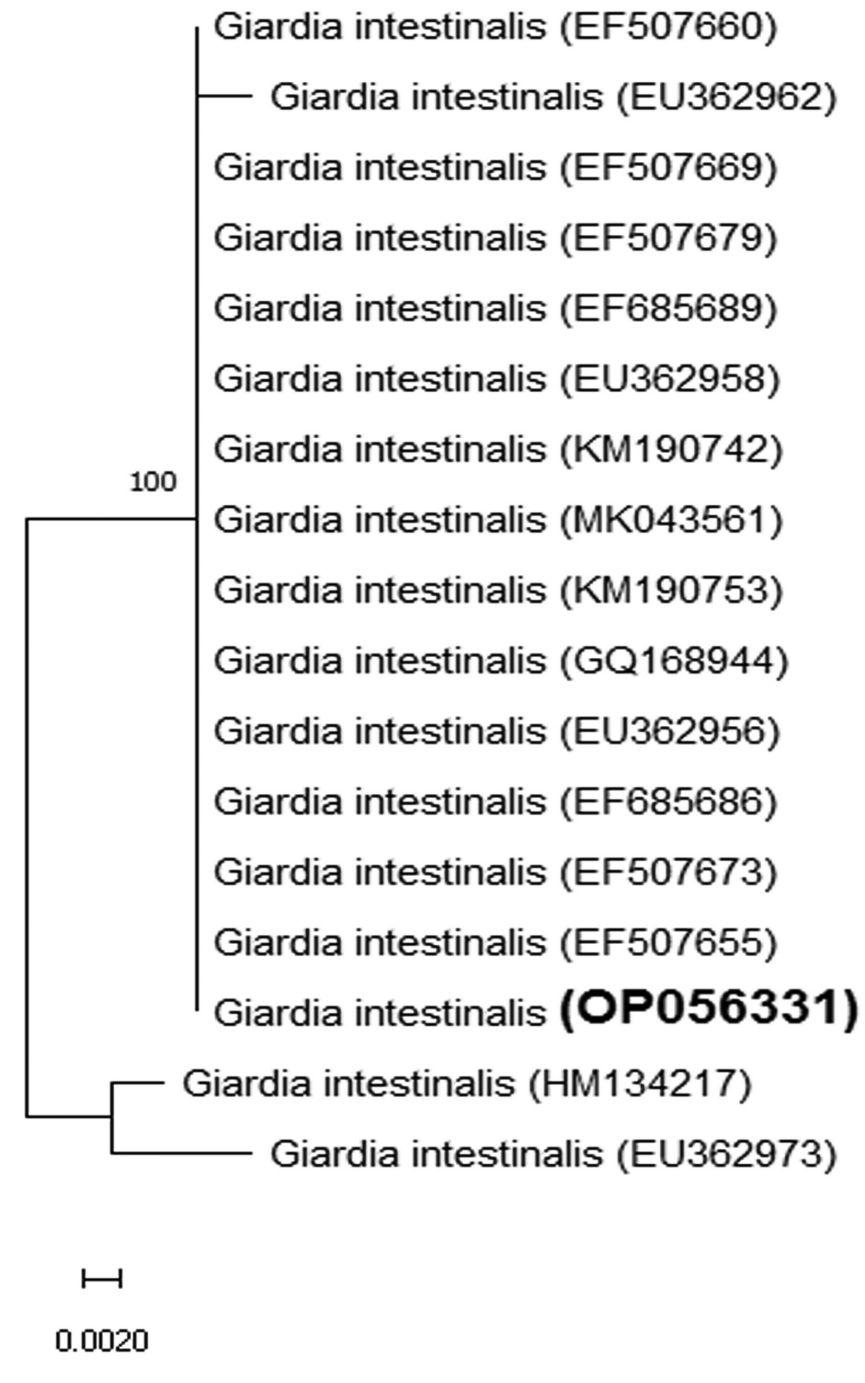
The phylogenetic tree constructed by BEAST. Bootstrap values are indicated at the branch position. OP056331 (the present study) was shown in bold.

The phylogenetic tree showed that the present isolate was located in the same clade with previously registered *G. duodenalis* isolates in Genbank (OP056331) (Figure 2). Molecular and sequence analysis confirmed the present isolate as genotype B of *G. duodenalis*.

### 3.3 Cyst Shedding

**FIGURE 3:**
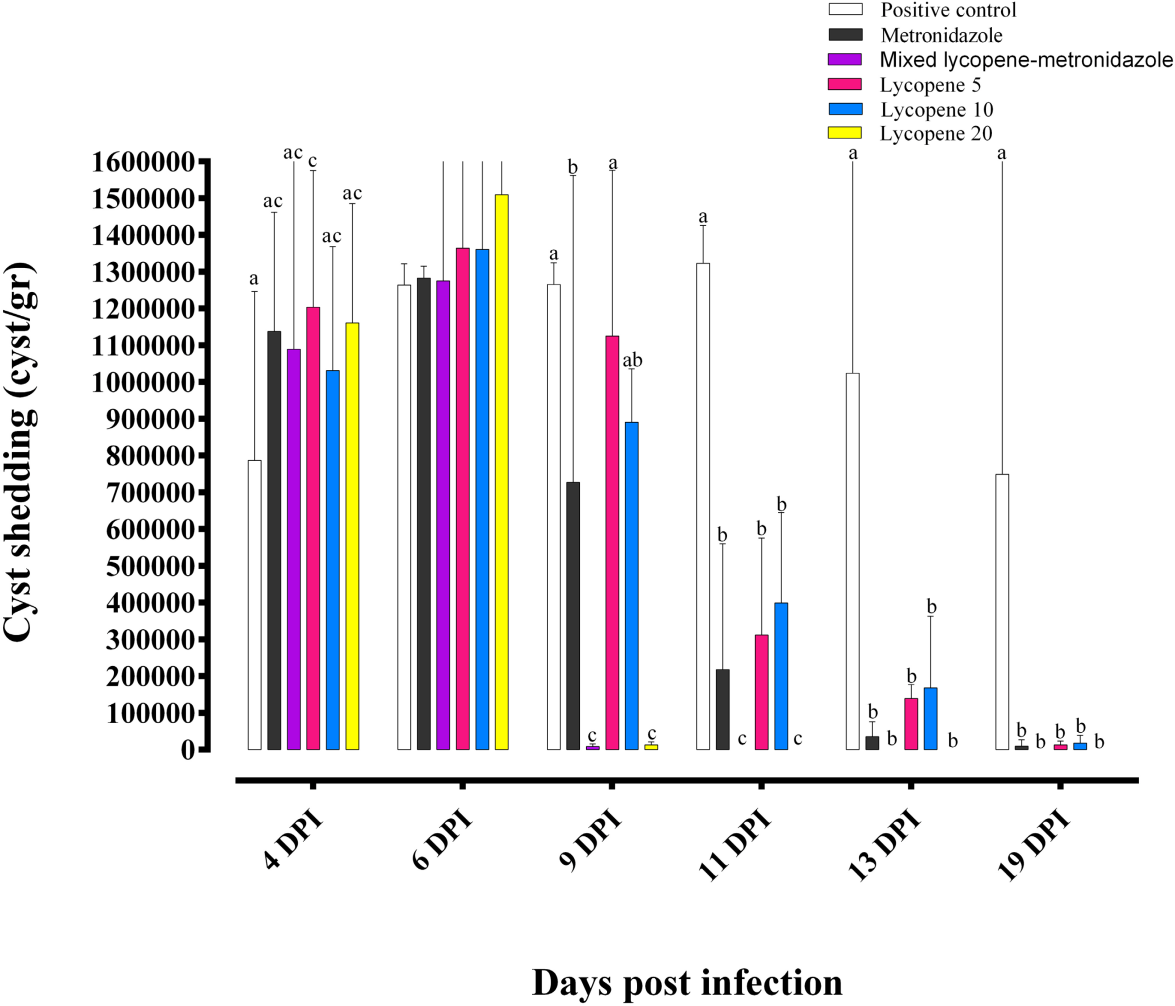
Rate of cyst shedding in different groups (*n* = 15) (mean ± SE). Dissimilar letters indicate a significant difference (*p* < 0.05).

Cyst shedding was observed in all groups on the fourth day after infection. On the sixth day after infection, the cyst shedding increased with no significant difference among the groups. The highest rate was observed on 9 days post‐infection in the positive control, lycopene 5 and 10, and the lowest observed in mixed lycopene‐metronidazole. On 9, 11, 13, and 19 days post‐infection, the shedding rate decreased in all treatment groups. The mice in groups lycopene 20 and mixed lycopene‐metronidazole had no cyst shedding from the 11^th^ day of infection (Figure 3).

### 3.4 Weight Changes

The highest weight was observed in the metronidazole group 9 days post‐infection (*p* < 0.05). Although the lowest weight was observed in the positive group, there was no significant difference among groups from the 11^th^ day until the end of the experiment (Table 1).

**TABLE 1:** Mean ± SE of weight (g) and percentage of weight changes (%) in mice with experimental giardiasis.

Sampling days												
Groups	4 Days post‐infection		6 Days post‐infection		9 Days post‐infection		11 Days post‐infection		13 Days post‐infection		19 Days post‐infection	
	Mean ± SE	%	Mean ± SE	%	Mean ± SE	%	Mean ± SE	%	Mean ± SE	%	Mean ± SE	%
Negative control	17.90 ± 0.69^b^	0.92	19.06 ± 0.94^b^	6.48	18.22 ± 0.73^b^	2.50	19.05 ± 0.96^ac^	5.93	19.45 ± 0.94^a^	6.92	19.29 ± 0.85^a^	8.15
Positive control	16.45 ± 0.21^c^	−6.42	17.64 ± 0.16^cd^	−1.67	18.67 ± 0.22^b^	5.84	16.93 ± 0.53^b^	−1.40	15.45 ± 3.10^a^	8.70	16.09 ± 3.22^a^	12.90
Metronidazole	19.62 ± 0.60^a^	0.00	20.64 ± 0.60^a^	5.31	20.96 ± 0.85^a^	6.43	20.10 ± 0.98^a^	1.88	19.65 ± 1.36^a^	−1.40	21.34 ± 1.56^a^	7.00
Lycopene 5 mg/kg	18.07 ± 0.32^b^	2.20	18.60 ± 0.37^bc^	4.73	18.26 ± 0.53^b^	2.75	18.03 ± 0.61^bc^	1.32	17.14 ± 0.60^a^	5.60	17.37 ± 0.36^a^	6.97
Lycopene 10 mg/kg	15.72 ± 0.24^c^	−0.26	16.47 ± 0.39^d^	4.47	17.47 ± 0.36^b^	10.89	17.60 ± 0.38^bc^	11.70	19.13 ± 0.27^a^	21.43	16.91 ± 0.39^a^	7.28
Lycopene 20 mg/kg	17.82 ± 0.42_b_	−2.22	18.49 ± 0.38^bc^	1.25	17.82 ± 0.43^b^	1.05	17.61 ± 0.43^bc^	1.32	16.76 ± 0.60^a^	−1.44	17.28 ± 0.43^a^	1.69
Mixed lycopene‐metronidazole	16.77 ± 0.27^bc^	−1.78	17.08 ± 0.37^d^	0.00	17.85 ± 0.55^b^	5.78	17.66 ± 0.48^bc^	4.67	19.12 ± 0.73^a^	11.42	19.02 ± 0.70^a^	10.91

*Note:* Dissimilar letters indicate a significant difference (*p* < 0.05). The positive and negative percentages show increased and decreased changes, respectively.

### 3.5 Histopathological Evaluation

**FIGURE 4:**
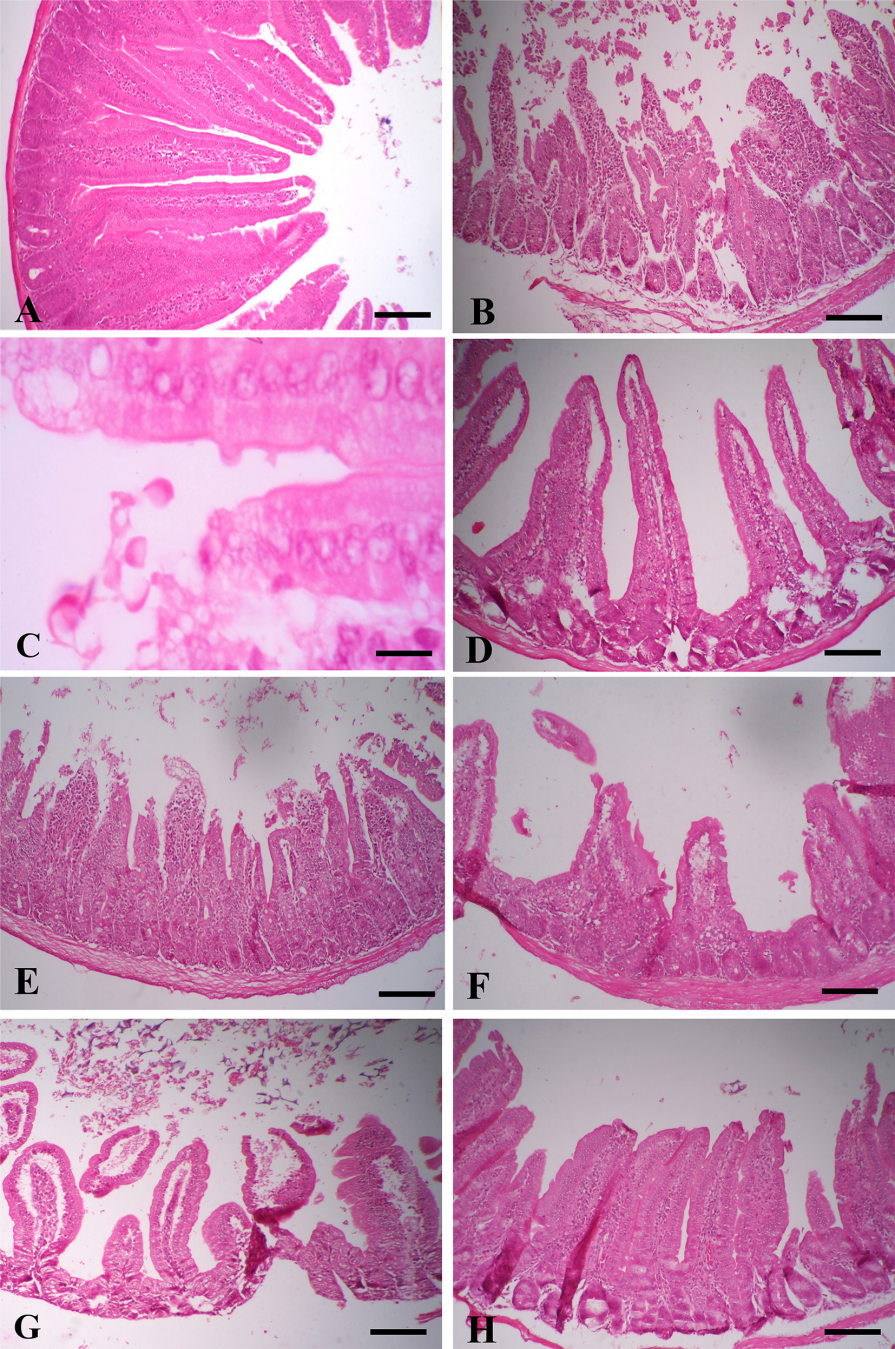
Histopathological evaluation of duodenal sections. (A) Negative control group: No lesion. Scale bar = 100 μm; (B) Positive control group: Shortening of villi and inflammatory cells infiltration in duodenal mucosa. Scale bar = 100 μm; (C) Positive control group: *Giardia duodenalis* trophozoites adherent to the intestinal villus and in the lumen. Scale bar = 10 μm; (D) Metronidazole group: Similar to normal structure. Scale bar = 100 μm; (E) 5 mg/kg lycopene group: Shortening of villi and inflammatory cells infiltration in the intestinal mucosa. Scale bar = 100 μm; (F) 10 mg/kg lycopene group: Shortening of villi. Scale bar = 100 μm; (G) 20 mg/kg lycopene group: Shortening of villi. Scale bar = 100 μm; (H) Mixed lycopene‐metronidazole group: Very mild shortening of villi. Scale bar = 100 μm. H&E.

There were no lesions in tissue sections of the negative control group (Figure 4A), while in the positive control group, mild shortening of villi and inflammatory cell infiltration with trophozoites adherent to the villus and in the lumen were observed in 7 out of 15 mice (Figure 4B,C).

In the metronidazole group, tissue structure was near‐normal architecture, and very mild shortening of villi was observed in 2 out of 15 mice (Figure 4D). In the treated groups with 5, 10, and 20 mg/kg lycopene, shortening of villi and inflammatory cell infiltration were observed in 5, 5, and 2 out of 15 mice, respectively. The severity of lesions was mild to moderate in 5 mg/kg lycopene and mild in others (Figure 4E–G). In the mixed lycopene‐metronidazole group, there was a shortening of villi in 2 out of 15 mice, which was milder compared to other groups (Figure 4H).

### 3.6 Antioxidant Status

The antioxidant status of duodenal tissues is shown in Table 2. There was no significant difference in both TAC and MDA between the positive control group and others in 6 day post infection.

**TABLE 2:** TAC and MDA level of deoudenal tissue in experimental giardiasis (mean ± SE).

Groups	6 Days post infection		13 Days post infection		19 Days post infection	
	TAC (μmol/L)	MDA (μmol/L)	TAC (μmol/L)	MDA (μmol/L)	TAC (μmol/L)	MDA (μmol/L)
Negative control	—	—	—	—	12.86 ± 1.42^b^	2.35 ± 0.62^c^
Positive control	5.38 ± 0.29^a^	44.21 ± 2.51^a^	4.29 ± 0.84^a^	53.61 ± 7.87^a^	4.21 ± 0.68^d^	53.74 ± 9.08^a^
Metronidazole	6.10 ± 0.62^a^	41.88 ± 1.28^a^	16.54 ± 7.15^b^	1.63 ± 0.87^b^	16.41 ± 3.51^a^	2.36 ± 0.34^c^
Lycopene 5 mg/kg	5.81 ± 0.19^a^	42.43 ± 0.75^a^	7.97 ± 1.15^a^	5.96 ± 1.05^b^	4.94 ± 0.51^d^	24.27 ± 3.59^b^
Lycopene 10 mg/kg	5.78 ± 0.46^a^	43.60 ± 1.38^a^	8.30 ± 1.88^a^	5.67 ± 2.06^b^	4.78 ± 0.51^d^	22.46 ± 0.73^b^
Lycopene 20 mg/kg	6.01 ± 0.56^a^	43.37 ± 1.40^a^	8.65 ± 1.03^a^	4.94 ± 1.23^b^	8.68 ± 0.76^c^	5.57 ± 2.87^c^
Mixed lycopene‐metronidazole	5.73 ± 0.74^a^	43.53 ± 1.54^a^	10.25 ± 0.91^a^	3.22 ± 0.47^b^	9.97 ± 0.76^bc^	2.92 ± 0.77^c^

*Note:* Dissimilar letters indicate a significant difference (*p* < 0.05).

Abbreviations: MDA, malondialdehyde; TAC, total antioxidant capacity.

On day 13 post‐infection, the highest amount of TAC was seen in the metronidazole group with a significant difference from the positive control group. Although the treatment led to an increase in TAC in other groups, there was no significant difference in the positive control. In all treatment groups, the level of MDA significantly decreased compared with the positive control and the lowest amount was observed in the metronidazole group.

On day 19 post‐infection, the treatment significantly increased the amount of TAC except for the 5 and 10 mg/kg lycopene groups. The level of MDA significantly decreased in all treatment groups in comparison with the positive control.

## 4 Discussion

*Giardia duodenalis* infects about one billion people worldwide annually (Ansell et al. 2015). Metronidazole is a drug of choice used to treat *Giardia* infections, but it has severe side effects and intolerance (Hill et al. 2005; Mørch and Hanevik 2020). In addition, the treatments sometimes are not effective due to drug resistance (Amer, Mossallam, and Mahrous 2014).

Tomatoes and tomato products contain lycopene (Górecka et al. 2020). It is a protective agent against various chronic diseases by reducing oxidative damage (Chauhan et al. 2011). A few studies have been performed on the effect of lycopene on some parasites, including *Plasmodium falciparum* (Agarwal et al. 2014), *Trypanosoma cruzi* (Junior et al. 2013), and *Plasmodium berghei* (Iswari et al. 2016).

Molecular diagnosis and assemblage identity can be used to classify the zoonotic and nonzoonotic *Giardia* isolates (Maloney, Molokin, and Santin 2020; Minvielle et al. 2008; Rayani et al. 2017; Saeedi, Jonaidi Jafari, and Salehzadeh 2018; Wilke and Robertson 2009). *Giardia lamblia* isolates were classified into seven assemblages from which A and B infect humans (Maloney, Molokin, and Santin 2020; Aloisio et al. 2006). Our isolate was identified as the assemblage B by sequencing of the GDH gene.

On the fourth day post‐infection, the cyst shedding started in all groups with an increasing trend until 13 days post‐infection. This finding is in accordance with Roberts‐Thomson et al. (1976), who reported the peak of cyst shedding from 5 to 14 PID and spontaneous shedding stop from 21 to 28 in *G. muris* infection. Our results revealed that the mice treated with metronidazole and mixed lycopene‐metronidazole had the lowest cyst‐shedding rate. The mechanism by which lycopene reduces cyst shedding can be due to its direct effect on the trophozoites, the changes in cyst formation, or the antioxidant capacity of intestinal tissue, which requires more studies.

The highest weight of mice was for the metronidazole group and the lowest for the positive control. Despite a significant difference between these groups in the first days of treatment, no significant difference was observed between the groups from the seventh day after treatment until the end of the study. A severe weight loss due to malabsorption syndrome in ovine (Aloisio et al. 2006) and murine giardiasis (Roberts‐Thomson et al. 1976) has been reported. The present results were in contrast with their finding probably due to shorter experiment period.

Diffuse shortening of the intestinal villi is considered a significant lesion of giardiasis, which reduces the mucosal surface to absorb nutrients, minerals, and etc. (Allain et al. 2017). In the present study, the tissue sections from the mice treated with metronidazole as a standard drug almost had a normal structure with a very mild shortening of villi. The treatment group with mixed lycopene‐metronidazole showed the least lesions compared with the positive control. The severity of the lesions decreased in the groups treated with doses of 5, 10, and 20 lycopene, respectively. Shortened villi, reduction in villus/crypt ratios, inflammatory cell infiltration, and hyperplasia of goblet cells have been also reported in *Giardia duodenalis* infection (Allain et al. 2021; Scott et al. 2000; Ventura et al. 2017). In the present study, although the same diet was applied in all experimental groups, Allain et al. (2021) showed that a diet rich in fat aggravates *Giardia* infection and can promote its pathogenesis. In addition, it has been suggested that a high‐fat diet elevates the severity of the disease by the persistence of *Giardia* and microflora alterations (Allain and Buret 2020).

TAC refers to a set of compounds that can protect biological systems against the harmful effects of reactive oxygen and nitrogen species (Fraga, Oteiza, and Galleano 2014; Rubio et al. 2016). The highest TAC was seen in metronidazole and mixed lycopene‐metronidazole groups. Several attempts have been directed to show consuming tomato extract rich in lycopene promotes antioxidant status (Bandeira et al. 2017; Bhatia, Singh, and Koul 2018; Dai et al. 2015; Jhou et al. 2017). A diet rich in lycopene stimulates endogenous antioxidative enzymes (Subhash, Bose, and Agrawal 2007) and has protective effects on lipids, proteins, and DNA (Agarwal and Rao 2000; Yonar et al. 2020).

Lipid peroxidation is the main consequence of oxidative stress demonstrated by increasing the MDA level (Ayala et al. 2014). Under deoxynivalenol exposure, lycopene protected cells from oxidative stress by decreasing MDA levels and increasing antioxidant enzymes and expression of p‐Nrf2 (Wang et al. 2020). Also, the cardioprotective effects of lycopene have been showed by restoring the levels of MDA and antioxidant parameters to normal in comparison with tulathromycin‐treated mice (Abdel‐Daim et al. 2018). In this experiment, the MDA level significantly decreased in all treatment groups in comparison with positive control on 7 days post‐treatment. The present study showed that lycopene consumption can ameliorate *Giardia* infection probably through increasing antioxidant status and reducing lipid peroxidation. This finding is consistent with that of Ugbaja et al. (2021) who showed lycopene could improve neuroinflammation by decreasing oxidative stress and down regulation of the TLR4/NF‐κB‐p65 axis.

## 5 Conclusions

Lycopene could stop *Giardia* cyst shedding by an unknown mechanism. As the antioxidant indices improved following lycopene consumption, it seems that the antigiardial effect of lycopene in our study is related to its antioxidant properties. Nevertheless, whether the lycopene has a direct effect on *Giardia* or not and its probable mechanism remain to be investigated.

## Author Contributions

Study design: F.N., S.M.R.; Performing the study: J.P., S.N., H.N.; Analysis: F.N., S.M.R.; Preparing the manuscript: F.N., S.M.R., J.P.; All authors read and approved the final manuscript.

## Ethics Statement

This study was approved by the Animal Ethics Committee (AECs) of School of Veterinary Medicine, Shiraz University (permit: 97GCU1M1316), and all the animal experiments were performed with our institutional guidelines and regulations (dated 20 September 2013) and ARRIVE guidelines for reporting animal research as much as possible (https://arriveguidelines.org/).

## Conflicts of Interest

The authors declare no conflicts of interest.

## Data Availability

All data generated or analyzed during this study are included in this article.
